# Use of Wearable Devices to Augment Traditional Measurements of Postoperative Outcomes Following Total Joint Arthroplasty: Systematic Review

**DOI:** 10.2196/84671

**Published:** 2026-04-17

**Authors:** Robert Raymond Hall III, Alexander P Hamel, Pamela A Chan, Hua Zheng, David C Ayers

**Affiliations:** 1 Department of Orthopedics and Physical Rehabilitation, University of Massachusetts Chan Medical School, 55 Lake Avenue North, Worcester, MA, 01655, United States, 1 7733837577; 2 University of Massachusetts Chan School of Medicine, Worcester, MA, United States

**Keywords:** remote monitoring, wearable device, total joint arthroplasty recovery assessment, patient-reported outcome measures, mobility, osteoarthritis

## Abstract

**Background:**

Wearable devices enabling remote monitoring by surgeons of their patients have gained prominence around total joint arthroplasty (TJA), offering continuous patient data to identify those not meeting postoperative goals, thereby facilitating timely interventions. While multiple studies highlight the utility of these devices in tracking postoperative progress, a standardized approach to their application is lacking. This review aims to synthesize existing literature on the use of wearable device-tracked activity for monitoring TJA outcomes.

**Objective:**

We examined the current literature to evaluate how wearable devices are used in monitoring and improving patient rehabilitation and outcomes following TJA.

**Methods:**

A systematic review was conducted following Cochrane methodology. A literature search of all available literature was performed in April 2024 and identified 102 studies to undergo full-text review. Systematic reviews, duplicate papers, and theoretical papers were excluded. Ultimately, 35 studies met the selection criteria.

**Results:**

The review revealed that 32 of 35 (91.4%) studies used wearable devices to monitor step counts. A total of 21 (60%) studies incorporated joint-specific patient-reported outcome measures, though the specific measures varied. Further, 9 studies used standardized performance-based outcome measures, which also differed across studies. Finally, 7 (20%) studies collected sleep data; however, the methods and outcomes for sleep measurement were inconsistent among these studies.

**Conclusions:**

Remote monitoring via wearable devices offers a novel approach to tracking outcomes in TJA patients. Although the use of these devices in perioperative care is expanding, significant variability exists in the data reported across studies. Wearable monitoring is often integrated with patient-reported outcome measures and standardized functional assessments, yet the optimal data parameters that best correlate with established outcome metrics remain undefined. Additionally, data collected by wearables has not yet been shown to predict patient recovery or satisfaction. Further research is essential to refine these data parameters and the development of postoperative protocols that leverage wearable devices to enhance patient compliance and improve clinical outcomes.

## Introduction

Total hip arthroplasty (THA) and total knee arthroplasty (TKA) are currently among the most commonly performed surgeries in the United States, with significant growth expected by 2030. The number of TKAs performed annually is projected to reach 1 million, and the number of THAs is projected to reach 600,000 by 2030 [1]. Despite high patient satisfaction rates, 10% to 20% of TKA patients and 5% to 15% of THA patients are not satisfied after surgery, suggesting that current recovery assessments may not adequately address all aspects of patient care [2,3]. The variability of current methods of assessing total joint arthroplasty (TJA) recovery exists due to divergent guidelines, institutional policies, difficulty in obtaining data, and physician preferences. Most commonly, assessment of patient recovery has used patient-reported outcome measures (PROMs).

The adoption of wearable technology is on the rise, with 30%‐50% of adults in the United States using devices that track various health metrics [4,5]. Wearables can continuously monitor critical health parameters such as heart rate, physical activity, and sleep during the postoperative recovery phase, providing valuable data for assessing recovery outcomes in TJA patients. Wearable devices provide the opportunity to obtain data detailing the recovery of patients after TJA, improve patient compliance with rehabilitation programs, and enhance patient engagement through real-time feedback [6].

Previous studies demonstrate that wearable devices can enhance physical activity levels and offer insightful data throughout the postoperative recovery period [7-9]. Hameed et al found that remote monitoring of TKA patients improved outcomes in the early postoperative period [10].

Presently, there is a notable absence of standardized methods for interpreting the extensive data generated by wearable devices in the context of TJA outcomes. This systematic review aims to clarify how wearable devices can effectively measure TJA outcomes, focusing on metrics such as sleep quality, physical activity, and step counts. Data from both TKA and THA patients will be included to potentially yield more holistic insights into the application of wearable technology in joint replacement scenarios. The goal of this systematic review is to significantly contribute to ongoing discussions aimed at enhancing patient outcomes and satisfaction following TJA surgery by leveraging the innovative capabilities of wearable technology.

## Methods

### Search Strategy

A thorough systematic review was conducted according to the Cochrane methodology for systematic reviews as described in the Cochrane Handbook for Systematic Reviews of Interventions [11] (Multimedia Appendix 1). Our trial was registered with PROSPERO (registration number CRD420261346230). A literature search was conducted in Scopus, Medline, CINAHL, PubMed, CENTRAL, and Google Scholar databases in April 2024 using relevant search terms associated with the research question, “Can wearable device data be used to effectively augment postoperative patient-reported outcomes after total joint replacement?” (Multimedia Appendix 2). Literature searches were not limited by date of publication.

### Screening and Assessment of Eligibility

A total of 471 papers were identified from the databases or registers used, as well as 7 studies from the reference lists of other papers. Selection criteria included availability in English and full-text accessibility. Systematic reviews and theoretical papers were excluded. After the removal of 100 duplicates, the abstracts of 371 studies were screened for compliance with the criteria. Further, 269 studies were excluded during abstract screening due to a lack of adherence to selection criteria, resulting in 102 studies to undergo full-text review. Finally, 35 studies were identified from the full-text review that satisfied the selection criteria for inclusion in this systematic review.

### Data Extraction and Analysis

Data extraction of the 35 studies was performed on Covidence. Data extraction was originally performed independently by 2 reviewers, then reviewed for consensus. Extracted data included information regarding study identification, aims, study design and population, inclusion and exclusion criteria, data collection characteristics, interventions, accelerometer details and data, PROMs and quality of life assessments used, and study end points.

### Risk of Bias Assessment

Study quality and bias risk were assessed using Cochrane’s Risk of Bias tool for randomized trials (RoB-2) [12] and the Risk of Bias in Nonrandomized Studies of Interventions (ROBINS-I) tool [13]. RoB-2 includes 5 domains: the randomization process, deviations from the intended intervention, missing outcomes data, measurement of the outcome, and selection of the reported result. ROBINS-I evaluates 7 domains: confounding, participant selection, intervention classification, deviations from intended interventions, missing data, outcome measurement, and selection of reported results. RoB-2 categorized bias as low risk, some concerns, high risk, or no information, while ROBINS-I classified risk as low, moderate, serious, critical, or no information. A total of 2 authors independently assessed the study quality of each paper and resolved any concerns with thorough discussion. Traffic light plots of domains regarding risk of bias were generated using the risk-of-bias visualization tool [14] (Multimedia Appendix 3). Notably, 5 studies were funded by Zimmer Biomet [15-19], in 1 study [20], 360 Knee Systems supplied the wearable device, and in 1 study [21], the lead author was a creator of the MoveUp Application that was used to assess patient recovery.

### Study Quality

The quality of the studies was assessed using RoB-2 for the 5 randomized controlled trials (RCTs) and ROBINS-I for the 30 nonrandomized studies. RCT data are presented in Table 1, and nonrandomized data in Table 2. For overall risk of bias, 5 studies were characterized as low risk, 16 studies were ranked moderate risk or some concerns, 11 studies were ranked serious risk, and 3 studies were characterized as not enough information.

**Table 1.** Risk of bias assessment for randomized controlled trials using the Cochrane Risk of Bias tool.

Study ID	Bias due to randomization	Bias due to the deviations from intended intervention	Bias due to missing outcomes data	Bias due to outcome measurement	Bias in the selection of the reported result	Overall
Paxton et al, 2018 [22]	High	Low	Low	Low	Low	Some concerns
Van der Walt et al, 2018 [23]	Low	Low	Low	Low	Low	Low
Duong et al, 2023 [24]	Low	Low	Low	Low	Low	Low
Mehta et al, 2020 [25]	No information	Low	Low	Low	Low	Some concerns
Pechstein et al, 2023 [26]	Low	Low	Low	Low	Low	Low

**Table 2.** Risk of bias in Nonrandomized Studies of Interventions.

Study ID	Bias due to confounding	Bias due to participant selection	Bias in classification of intervention	Bias due to deviations from intended intervention	Bias due to missing data	Bias in measurements of outcomes	Bias in the selection of the reported result	Overall
Tang et al, 2021 [27]	Moderate	Moderate	Low	Low	Moderate	Low	Low	Moderate
Crizer et al, 2017 [28]	Moderate	Moderate	Low	Low	No Information	Low	Low	No Information
Caliskan et al, 2020 [29]	Moderate	Serious	Low	Low	Low	Low	Low	Serious
Brandes et al, 2011 [30]	Moderate	Serious	Low	Low	Moderate	Low	Low	Serious
Frimpong et al, 2020 [31]	Low	Serious	Low	Low	Serious	Low	Low	Serious
Höll et al, 2018 [32]	Low	Serious	Low	Low	No Information	Low	Low	No Information
Redfern et al, 2023 [15]	Moderate	Moderate	Low	Low	No Information	Low	Low	No Information
Komann et al, 2024 [33]	Low	Low	Low	Low	Serious	Low	Low	Serious
Bolander et al, 2024 [34]	Moderate	Moderate	Low	Low	Low	Low	Low	Moderate
Harding et al, 2014 [35]	Moderate	Moderate	Low	Low	Moderate	Low	Low	Moderate
Bendich et al, 2019 [36]	Moderate	Moderate	Low	Low	Serious	Low	Low	Serious
Twiggs et al, 2018 [20]	Moderate	Moderate	Low	Low	Moderate	Low	Low	Moderate
Bini et al, 2019 [37]	Moderate	Moderate	Low	Low	Serious	Low	Low	Serious
Shah et al, 2019 [38]	Moderate	Low	Low	Low	Moderate	Low	Low	Moderate
Frimpong et al, 2019 [39]	Moderate	Serious	Low	Low	Serious	Low	Low	Serious
Lebleu et al, 2021 [40]	Low	Moderate	Low	Low	Moderate	Low	Low	Moderate
Vitale et al, 2023 [41]	Moderate	Moderate	Low	Low	Low	Low	Low	Moderate
van Diemen et al, 2023 [42]	Moderate	Moderate	Low	Low	Low	Low	Low	Moderate
Gibian et al, 2023 [19]	Moderate	Moderate	Low	Low	Serious	Low	Low	Serious
Christensen et al, 2023 [16]	Moderate	Moderate	Low	Low	Low	Low	Low	Moderate
Sato et al, 2023 [17]	Moderate	Moderate	Low	Low	Low	Low	Low	Moderate
Fallahzadeh et al, 2023 [43]	Low	Moderate	Low	Low	Moderate	Low	Low	Moderate
Karas et al, 2020 [44]	Low	Low	Low	Low	Low	Low	Low	Low
Vitale et al, 2023 [41]	Moderate	Moderate	Low	Low	Low	Low	Low	Moderate
Lebleu et al, 2024 [21]	Low	Low	Low	Low	Low	Low	Low	Low
Harada et al, 2024 [45]	Low	Serious	Low	Low	Serious	Low	Low	Serious
Surmacz et al, 2024 [46]	Moderate	Low	Low	Low	Low	Low	Low	Moderate
Lyman et al, 2020 [47]	Low	Moderate	Low	Low	Serious	Low	Low	Serious
Pasqualini et al, 2024 [18]	Moderate	Moderate	Low	Low	Moderate	Low	Low	Moderate
Goeb et al, 2021 [48]	Low	Serious	Low	Low	Moderate	Moderate	Low	Serious

### Systematic Review Procedure and Narrative Synthesis Process

The literature identified for review demonstrated considerable variability in variables collected via wearable devices as well as outcomes reported. As such, a meta-analysis was not performed. Rather, a systematic review was performed according to the Synthesis Without Meta-Analysis in systematic reviews guideline [49]. Studies were grouped according to outcomes reported, as demonstrated in Table 3. The results, limitations, and data presentation from our synthesis are also presented in Table 3.

**Table 3.** Papers included in the systematic review grouped by measurements of interest.

Study ID	Aim of the study	Participants (n)	TKA?^a^ THA?^b^ both?	Device (placement)	Accelerometer data collected or assessed	Recovery assessments	Timeline	Summary of findings
Physical activity and PROMs^c^								
Bendich et al, 2019 [36]	Determine if perioperative patient-generated health data, such as steps and activity levels, are associated with early post-op PROMs.	22	Both	Fitbit Flex Google LLC; wrist)	Steps; physical activity; HR^d^	KOOS^e^ or HOOS^f^	Pre-op:^g^ N/A^h^ Post-op^i^: ≤6 weeks	Changes from pre-op to 6-weeks post-op levels steps are associated with improvements in PROMs. Only a weak association between absolute sensor values and PROM values after TJA^j^.
Bini et al, 2019 [37]	Predict early post-op TJA outcomes using wearable sensor data and machine learning.	22	Both	Fitbit Flex (wrist)	Steps; physical activity; HR; personalized activity intelligence; cadence; bounce	KOOS or HOOS, VR-12^k^	Pre-op: ≤4 weeks Post-op: ≤8 weeks	Moderate correlation between post-op activity levels (steps, minutes active, and metabolic output) and VR-12 PCS^l^ scores. No correlation between preoperative steps and PROMs.
Brandes et al, 2011 [30]	Measure change in physical activity and the relationship between objective function data and pain post-TKA.	53	Total knee	SAM^m^ step activity monitor (Cyma Corporation; ankle)	Steps	KSS^n^, SF-36^o^	Pre-op: N/A Post-op: ≤12 mo	Post-op physical activity is influenced by pre-op physical activity, though post-op physical activity at 12 mo is not correlated with KSS or SF-36 scores pre-op or post-op.
Caliskan et al, 2020 [29]	Identify accelerometer-recorded physical activity patterns in patients undergoing TKA in addition to ROM^p^, strength, and PROMs.	36	Total knee	ActiCal (Respironics Inc; wrist)	Steps; physical activity; energy expenditure	ROM; muscle strength; KSS, OKS^q^	Pre-op: ≤1 week Post-op: ≤6 mo	KSS, OKS, light and moderate physical activity, steps, and daily energy expenditure significantly improved from baseline to 6 months post-op, while knee flexion and extension strength and sedentary behavior did not improve.
Christensen et al, 2023 [16]	Describe the trajectory of post-TKA recovery based on PROMs and smartwatch physical activity.	1005	Total knee	Apple Watch (Apple Inc; wrist)	Steps; stairs; gait speed; gait asymmetry	KOOS JR^r^, EQ-5D	Pre-op: N/A Post-op: ≤12 mo	Perceived knee health and QoL^s^ scores increased from pre-op to 12 months post-op while following a maximal recovery trajectory in the first 3 months. Steps per day increased to pre-op levels by 1 month and followed a maximal recovery trajectory in the first 3 months, while stairs per day returned to baseline by 3 months and continued to improve beyond 6 months.
Crizer et al, 2017 [28]	Track daily step count during pre-op and post-op periods and compare with PROMs.	589	Both	Smartphone (N/A)	Steps	LEFS^t^	Pre-op: ≤4 weeks Post-op: ≤12 weeks	LEFS scores only weakly correlated with objective step count, with increasingly weaker correlation with time post-op. No clear correlation at 12 weeks post-op suggests PROMs may only provide part of the story. Physical activity recovery is most rapid in THA patients, with most surpassing baseline step counts by 5 weeks compared to 9 in TKA.
Duong et al, 2023 [24]	Compare a digital technology package with usual care in reducing pain among participants post-TKA.	102	Total knee	Fitbit Inspire (Google LLC; wrist)	Steps; physical activity; sleep	NRS^u^, PDI^v^, Sedentary Behavior Questionnaire, AQoL-8D, Patient Activation Measure, Modified Computer Self-Efficacy Scale, Change in pain intensity (Global Rating of Change)	Pre-op: N/A Post-op: ≤12 mo	Patient-reported physical activity (AAS^w^) significantly increases across all participants from baseline (10 days post-op) to 3, 6, and 12 months. Fitbit physical activity measures (ie, steps and sedentary time) do not see the same significant increases. Sleep duration and time in bed remain unchanged from 3 to 6 months, though they significantly decrease from 6 months post-op to 12 months. Digital technology group demonstrates reduced pain intensity and PDI scores and increased physical activity (step count and stepping time).
Fallahzadeh et al, 2023 [43]	Use wearable device data and immune profiling to quantify physical recovery in individuals following THA.	49	Total hip	ActiGraph (Amertis LLC; wrist)	Steps; physical activity; sleep	WOMAC^x^, SRS^y^	Pre-op: ≤5 days Post-op: ≤40 days	Activity capacity, sleep disruption, and sedentary activity are most predictive of time after surgery, while light, moderate, and overall activity are less predictive in the model. Objective physical recovery trajectory is significantly associated with SRS, but not WOMAC, suggesting that smartwatch data may be more associated with self-reported fatigue and ability to perform daily activities than pain and overall physical function. Pre-op activity levels are not associated with post-op recovery speed.
Frimpong et al, 2020 [31]	Assess whether changes in objectively measured physical activity were associated with PROMs before and after TKA.	89	Total knee	activPAL (PAL Technologies Ltd; thigh)	Steps; sleep; sedentary time; standing time; walking time	KOOS, OKS	Pre-op: ≤2 weeks Post-op: ≤6 mo	Despite significant improvements in self-reported perceived knee pain and functional ability (KOOS and OKS) 6 months post-TKA, changes do not correlate with improvements in objectively measured (activPAL) light-intensity and sedentary activity behaviors. Average activity behaviors worsened 6 weeks post-op compared to pre-op, though by 6 months post-TKA, activity behaviors had improved to at or beyond pre-op levels.
Gibian et al, 2023 [19]	Measure patient sleep using a wearable device following TKA and compare with PROMs.	110	Total knee	Fitbit Inspire (wrist)	Sleep	PSQI^z^, VAS^aa^	Pre-op: N/A Post-op: ≤90 days	Patient-reported sleep quality (very bad sleep) correlated well with 30-day VAS pain score, while sleep duration (monitored or patient-reported) did not correlate with any clinical measure and was not shown to be a useful metric in assessing TKA outcome.
Harada et al, 2024 [45]	Compare objective and subjective measurements of activity levels in patients undergoing THA pre-op and post-op.	42	Total hip	ActivPal3 (PAL Technologies Ltd; thigh)	Steps; upright time; sit-to-stand transitions	OHS^ab^, UCLA^ac^, FJS-12^ad^	Pre-op: N/A Post-op: ≤1 year	Steps, OHS, and UCLA significantly improve from pre-op to 3 months and 1 year post-op, and age and hip abductor muscle volume are significant predictors of post-op step counts, while pre-op UCLA score does not predict postoperative objective activity data.
Harding et al, 2014 [35]	Measure physical activity pre-op and (6 months) postarthroplasty and examine the proportion of people meeting the American Physical Activity Guidelines.	44	Both	ActiGraph GT1M (Amertis LLC≈; wrist)	Physical activity	NPRS^ae^, OHS/OKS, SF-12^af^, UCLA	Pre-op: N/A Post-op: ≤6 mo	No change in objectively measured physical activity after arthroplasty, despite significant improvements in PROMs post-THA/TKA. There were significant improvements in self-reported pain during activity, physical function, QoL, and in physical activity measured by the UCLA activity score after arthroplasty.
Höll et al, 2018 [32]	Measure activity levels before and after THA.	46	Total hip	StepWatch 3 (Modus Health LLC; ankle)	Steps; physical activity	HHS^ag^, WOMAC	Pre-op: ≤1 week Post-op: ≤3 mo	Gait cycles (1 gait cycle =approximately 2 steps) and time in moderate to vigorous physical activity did not increase from pre-op to 6 weeks, but did increase at 3 mo follow-up, while PROMs increased at both follow-up time points. Gait cycles per day did not correlate with PROMs at any time point.
Karas et al, 2020 [44]	Use patient-generated health data from a wearable device to predict recovery trajectories from lower limb surgeries.	196		Fitbit (Google LLC; wrist)	Steps; HR; sleep	Self-reported recovery time	Pre-op: ≤26 weeks Post-op: ≤26 weeks	Daily steps return to baseline in ≈12 weeks post-op, while 95th percentile HR returns to baseline in roughly 4 weeks. Sleep efficiency does not follow a specific trajectory pre-op or post-op, though there are significant reductions in sleep efficiency from baseline up to 24 weeks post-op. Long-term recovery can be accurately predicted on an individual level 1 month after surgery. Further, 12 weeks pre-op and 26 weeks post-op trajectories of daily behavioral measurements (steps sum, HR, and sleep efficiency score) can capture activity changes relative to an individual’s baseline.
Komann et al, 2024 [33]	Assess the relationship between postoperative physical activity and PROMs.	55 (TKA)	Total knee	ActiGraph wGT3X (Amertis LLC; wrist)	Steps	BPI^ah^, IPO^ai^ Questionnaire	Pre-op: N/A Post-op: ≤7 days	There is no statistically significant correlation between steps and average pain intensity post-op.
Lebleu et al, 2021 [40]	Assess physical activity recovery post-op and determine perioperative features that could help predict physical activity recovery at 3 mo with telerehabilitation intervention.	146	Both	Nokia Go (Nokia Corporation; wrist)	Steps	KOOS or HOOS	Pre-op: ≤1 week Post-op: ≤3 mo	Patients receiving telerehabilitation reached pre-op step count at 7 weeks post-op. Change in step counts post-op at 3, 6, and 11 weeks could be predicted by pre-op step count and post-op length of time on crutches. Further, 3-month step increases are additionally associated with pre-op symptoms level, while early (3‐6 weeks) step count improvements are associated with pre-op QoL.
Lyman et al, 2020 [47]	Investigate the feasibility of mobile technology to collect daily step data and biweekly PROMs to track TJA recovery: (1) what proportion of patients provided full 6-month follow-up step and PROMs information, (2) whether THA and TKA patients recovered differently, (3) if new patterns of recovery could be discovered, and (4) how well step counts and PROMs scores were correlated.	267	Both	Smartphone accelerometer (N/A)	Steps	NRS, HOOS JR or KOOS JR	Pre-op:≥2 days Post-op: ≤6 mo	Recovery was faster by all metrics in THA than in TKA patients, correlations between step counts and PROMs were modest, and 2 distinct clusters of recovery could be described using step counts.
Pasqualini et al, 2024 [18]	Investigate the correlation between pain and KOOS JR with step and stair flight counts in patients undergoing TKA.	2333	Total knee	Apple Watch (Apple Inc; wrist)	Steps; stairs	KOOS JR, NRS	Pre-op:≥14 days Post-op: ≤3 mo	Step count is correlated with NRS and KOOS JR, both pre-op and at 1 and 3 mo post-op in TKA patients, while stair flight count is significantly correlated with PROMs pre-op and at 1 month, though no longer associated at 3 months.
Redfern et al, 2023 [15]	Investigate objective measures and PROMs as a function of preoperative PA^aj^ levels in TKA patients.	1941	Total knee	Apple Watch (Apple Inc; wrist)	Steps; physical activity	EQ-5D-5L, EQ-VAS, KOOS JR, KSS, NRS	Pre-op: ≤2 weeks Post-op: ≤12 mo	Patients in the 2 middle quartiles of wearable device-measured pre-op step counts recovered activity with a modest increase in step counts 1 year after surgery, while those in the highest quartile of activity did not return to preoperative levels at any interval through 1 year. The lowest-activity participants reported greater improvements in joint function, pain, and health-related QoL. Despite this, satisfaction scores did not vary between groups.
Sato et al, 2023 [17]	Identify the trajectory of physical activity and PROMs after THA.	1898	Total hip	Apple Watch (Apple Inc; wrist)	Steps; stairs; walking asymmetry; gait speed	HOOS JR, EQ-5D	Pre-op: N/A Post-op: ≤1 year	At all post-op time points, THA patients see statistically significant increases in steps, stair flights, walking asymmetry, and gait speed, though only steps and stair flights were clinically significant at 3, 6, and 12 months. Meanwhile, PROM improvements are clinically significant at all time points. Greatest PROM improvements were reached by 1 mo and physical activity by 3 months.
Shah et al, 2019 [38]	Determine the frequency and timeline of wearable device activity data that correlate with early PROMs following TJA.	22	Both	Lumo Lift (Lumo Inc, ND^ak^)	Steps; bounce; braking; drop or roll; pelvic rotation or yaw; or pelvic tilt or pitch	VR-12 PCS, HOOS or KOOS	Pre-op: N/A Post-op: 42 days	Raw wearable device data predicts 6-week post-op outcomes significantly better than 24-hour summarized data does, and clinical outcomes may be predictable by wearable device data as early as 1‐2 weeks post-op. Increasing sampling frequency above the standard 24-hour average provided by consumer-grade activity sensors improves the ability of machine learning algorithms to predict 6-week PROMs.
Surmacz et al, 2024 [46]	Determine the association between pain and PROMs or objective mobility data.	327	Both	Apple Watch (Apple Inc; wrist)	Steps; gait speed; gait asymmetry	HOOS or KOOS JR	Pre-op: ≤30 days Post-op: ≤90 days	Pain scores are associated with gait speed at all 3 time points, whereas step count was not significantly associated with pain at any time point.
Tang et al, 2021 [27]	Compare accelerometer-measured physical activity and sleep to PROMs following THA.	41	Total hip	Fitbit Flex (wrist)	Steps; sleep	HOOS JR	Pre-op: N/A Post-op: ≤3 mo	No correlation between accelerometry-measured activity level nor sleep and functional PROM scores in the early follow-up period (within 3 months) following THA. Average daily steps and minutes slept pre-op were similar to average daily steps and minutes slept 3 months post-op. Weak negative correlation between average steps pre-op and pre-op HOOS JR. Significant improvement in patients’ average daily sleep occurs between 1‐2 weeks and 1 month post-op.
Twiggs et al, 2018 [20]	Assess pre-op and early post-op physical activity in TKA patients and determine benchmarks for expected activity.	91	Total knee	Fitbit Flex (wrist)	Steps	SF-12, KOOS	Pre-op: ≤2 weeks Post-op: ≤6 weeks	SF-12 PCS and KOOS ADL were significantly correlated with step counts at all 3 time points. KOOS QoL was significantly associated with post-op 6-week step count, while KOOS pain was significantly associated with post-op day 2‐4 step count and pre-op step count. Pre-op step count is both strongly significantly correlated to and not statistically different from 6-weeks post-op step count. BMI and SF-12 PCS scores may also be predictive of post-op recovery.
Van der Walt et al, 2018 [23]	Determine if feedback from a wearable device improves activity levels over 6 weeks post-op following TJA.	163	Both	Garmin Vivofit 2 (Garmin Ltd; wrist)	Steps	KOOS, EQ-5D	Pre-op: ≤2 weeks Post-op: ≤6 mo	Feedback from wearable activity trackers increases daily activity in the acute post-op phase and at 6 months. The greatest increase in average daily steps post-op for both groups occurs during the 1‐ to 2-week period. Most patients surpass their pre-op step counts after 6 weeks post-op. Patients who received feedback from a commercial wearable device activity tracker with a daily step goal had significantly higher activity levels after hip or knee arthroplasty over 6 weeks and 6 months than those who did not receive feedback.
Vitale et al, 2021 [50]	Assess the objective physical activity and rest-activity daily rhythm in TJA patients.	20	Both	Actiwatch 2 (Amertis LLC; wrist)	Physical activity; RAR^al^	FIM, Barthel Index	Pre-op: 1 day Post-op: 10 days	Patients restored and maintained a significant and physiological RAR beginning 2 days post-op. Improvements can be seen in physical activity, BI^am^, FIM^an^, TUG^ao^, pain, and 10MWT^ap^ as early as 10 days post-op.
Physical activity and performance-based measures								
Paxton et al, 2018 [22]	Evaluate the feasibility of a physical activity feedback intervention for patients after TJA.	45	Both	Fitbit Zip (Google LLC; waist) and GT3X ActiGraph Activity Monitor (wrist) (Amertis LLC)	Steps; physical activity	6MWT, TUG; gait speed (m/s)	Pre-op: N/A Post-op: ≤12 weeks	Intervention participants (physical activity feedback) improved daily step count from baseline to 12 weeks by 20% compared to 9% for the control group. 6MWT increased 20% and 7% while TUG improved 1% and 4% for intervention and control groups, respectively. Goal setting needs to be adjusted to set realistic improvements.
Pechstein et al, 2023 [26]	Relate walking endurance with physical activity post-TKA and determine if this relationship differs between above vs below age-normative endurance.	109	Total knee	ActiGraph GT3X (Amertis LLC; waist)	Steps; physical activity	6MWT	Pre-op: N/A Post-op: ≤12 mo	Potential dose-response relationship between walking endurance and PA in adults after TKR^aq^, which may be present in those below age-normative walking endurance standards but absent in those with restored walking endurance.
Physical activity, PROMs, and performance-based measures								
Bolander et al, 2024 [34]	Characterize presurgical behavior using wearable devices and compare to post-op.	82	Total knee	TracPatch Duo (TracPatch Health LLC, knee)	Steps; flexion or extension	ROM; VAS	Pre-op: ≤6 days Post-op: ≤50 days	Daily step count and flexion return to baseline at 6- to 7-weeks post-op. Extension is regained within 1 week post-op. Pain scores improve past the pre-op baseline by 3 weeks post-op.
Frimpong et al, 2019 [39]	Describe changes in objectively measured physical activity and sedentary behavior in patients undergoing TKA.	79	Total knee	ActiGraph GT3X (wrist)	Steps; physical activity; sleep	ROM, UCLA, WOMAC	Pre-op: ≤7 days Post-op: ≤6 mo	While PROMs and knee ROM significantly improve from baseline at both 6 weeks and 6 months post-TKA, steps per day decrease significantly at 6 weeks, then significantly increase beyond baseline between 6 weeks and 6 months. Further, overall sedentary time and time spent in light physical activity improve significantly at 6 months, while moderate to vigorous physical activity does not. Clinically, functional improvements in patients post-TKA may be assessed by objectively measuring changes in low-intensity activity behaviors.
Goeb et al, 2021 [48]	Assess early outcomes of the posterior THA approach with modified precautions with the aid of wrist-based activity trackers.	82	Total hip	Letscom (Letscom International Corporation, Ltd; wrist)	Steps	Pain medication use, return to work or driving, assistive device use, HOORS JR, LEFS, pain	Pre-op: ≤1 week Post-op: ≤6 weeks	Patients consistently increased their average daily step count over the first 6 weeks post-op in a nearly linear fashion, with a slight reduction in the rate of improvement in weeks 5 and 6. Improvement in steps significantly correlated with changes in HOOS JR, LEFS, and pain scores over time.
Lebleu et al, 2024 [21]	Examine the incorporation of unsupervised evaluations and continuous monitoring with an activity tracker in the rehabilitation of total joint surgery.	1144	Both	Garmin Vivofit 4 (Garmin Ltd; wrist)	Steps; physical activity	6MWT, OKS, FJS^ar^, HOOS or KOOS, UCLA, EQ-5D	Pre-op: ≤1 week Post-op: ≤3 mo	Cadence measurements (ie, P6MC^as^ and P1M^at^) may offer more stable indicators of gross motor function compared to the number of steps per day. Patients typically resumed their pre-op activity levels 6‐10 weeks after undergoing surgery. Age significantly affects steps per day and cadence in TKA/THA. Patients who met MCID^au^ for FJS at 3 months surpassed baseline physical activity metrics, particularly light intensity activity, between 25 and 50 days post-op. Patients who did not meet the MCID for FJS were less likely to surpass baseline PA.
Mehta et al, 2020 [25]	Evaluate the effect of activity monitoring and bidirectional text messaging on the rate of discharge to home and clinical outcomes in TJA patients.	242	Both	Withings (Apple Inc, ND)	Steps	TUG, rehospitalizations, ED^av^ visits, PT visits, pain scale	Pre-op: N/A Post-op: ≤6 weeks	No significant increase in step count in those receiving remote monitoring plus gamification and social support compared to remote monitoring alone. Activity monitoring might have encouraged increases in step count, which may have improved the recovery function. Mean increase in daily step counts of 833 was found in intervention and control groups from 2 to 6 weeks post-op.
van Diemen et al, 2023 [42]	Assess the relationship between mitochondrial function at baseline and recovery of mobility following TKA.	30	Total knee	Nokia Go (Nokia Corporation; wrist)	Steps	TUG, quadriceps strength, grip strength, KOOS	Pre-op: ≤2 weeks Post-op: ≤6 mo	Pre-op mitochondrial function, grip strength, and steps correlate significantly with post-op step recovery. Post-op activity correlated significantly with KOOS-ADL^aw^ and TUG, but not with quadriceps or grip strength. Median recovery of steps was 101 steps per week (32-392 steps). Per 1 step of activity, recovery increases by 0.02 steps/weeks (*P*=.005).
Sleep								
Duong et al, 2023 [24]	Compare a digital technology package with usual care in reducing pain among participants following TKA.	102	Total knee	Fitbit Inspire (wrist)	Steps; physical activity; sleep	NRS, Pain Disability Index, Sedentary Behavior Questionnaire, AQoL-8D, Patient Activation Measure, Modified Computer Self-Efficacy Scale, Change in pain intensity (Global Rating of Change)	Pre-op: N/A Post-op: ≤12 mo	While patient-reported PA (AAS) significantly increases across all participants from baseline (10 days post-op) to 3, 6, and 12 months, Fitbit-recorded physical activity measures, such as steps and sedentary time, do not see the same significant increases, although there was no correlation analysis between the outcomes. Sleep duration and time in bed appear to remain unchanged from 3 to 6 months, though they significantly decrease from 6 to 12 months following TKA. The intervention significantly reduced pain intensity, improved the Pain Disability Index score, and increased physical activity (step count and stepping time).
Fallahzadeh et al, 2023 [43]	Use wearable device data and immune profiling to quantify physical recovery in individuals following THA.	49	Total hip	ActiGraph (wrist)	Steps; physical activity; sleep	WOMAC, SRS	Pre-op: ≤5 days Post-op: ≤40 days	Activity capacity, sleep disruption, and sedentary activity are most predictive of time after surgery, while light, moderate, and overall activity are less predictive in the model. Objective physical recovery trajectory is significantly associated with SRS, but not WOMAC, suggesting that smartwatch-derived data may be more associated with self-reported fatigue and ability to perform daily activities than pain and overall physical function. Pre-op activity levels are not associated with postoperative recovery speed.
Frimpong et al, 2020 [31]	Assess whether changes in objectively measured physical activity were associated with PROMs before and after TKA.	89	Total knee	activPAL (thigh)	Steps; sleep; sedentary time; standing time; walking time	KOOS, OKS	Pre-op: ≤2 weeks Post-op: ≤6 mo	Despite significant improvements in self-reported perceived knee pain and functional ability 6 months post-TKA, these changes do not correlate with improvements in objectively measured (using activPAL) light-intensity and sedentary activity behaviors. On average, activity behaviors worsened 6 weeks after surgery compared to preoperative levels, though by 6 months post-TKA, activity behaviors either were similar to pre-op levels or had improved beyond pre-op levels, such as in the case of step measures.
Gibian et al, 2023 [19]	Measure patient sleep using a wearable device following TKA and compare with PROMs.	110	Total knee	Fitbit Inspire (wrist)	Sleep	PSQI, VAS	Pre-op: N/A Post-op: ≤90 days	Patient-reported sleep quality (very bad sleep) correlated well with VAS pain score at 30 days, while sleep duration (monitored or patient-reported) did not correlate with any clinical measure and was not shown to be a useful metric in assessing TKA outcome.
Karas et al, 2020 [44]	Use patient-generated health data from a wearable device to predict recovery trajectories from lower limb surgeries.	196		Fitbit (wrist)	Steps; HR; sleep	Self-reported recovery time	Pre-op: ≤26 weeks Post-op: ≤26 weeks	Daily steps return to baseline in ≈12 weeks post-op, while 95th percentile HR returns to baseline in roughly 4 weeks. Sleep efficiency does not follow a specific trajectory pre-op or post-op, though there are significant reductions in sleep efficiency from baseline up to 24 weeks post-op. Long-term recovery can be accurately predicted on an individual level 1 month after surgery. Further, 12 weeks pre-op and 26 weeks post-op trajectories of daily behavioral measurements (steps sum, HR, and sleep efficiency score) can capture activity changes relative to an individual’s baseline.
Tang et al, 2021 [27]	Compare accelerometer-measured physical activity and sleep to PROMs following THA.	41	Total hip	Fitbit Flex (wrist)	Steps; sleep	HOOS JR	Pre-op: N/A Post-op: ≤3 mo	No correlation between accelerometry-measured activity level or sleep and functional PROM scores in the early follow-up period (within 3 months) following TJA (THA). Average daily steps and minutes slept pre-op were statistically like average daily steps and minutes slept 3 months post-op. Weak negative correlation between average steps pre-op and pre-op HOOS JR. Significant improvement in patients’ average daily sleep occurs between 1‐2 weeks and 1 month post-op.
Vitale et al, 2023 [41]	Assess wearable device-derived sleep characteristics and pain scores in patients hospitalized for recovery from TJA.	20	Both	Actiwatch 2 (Amertis LLC; wrist)	Sleep	TUG, 10MWT, PSQI, VAS	Pre-op: 1 day Post-op: 10 days	ActiGraph parameters of sleep quantity and timing did not differ from pre-op to 10-day post-op periods in TJA patients, though sleep efficiency and quality drastically decreased the first night after the surgery before improving in the following 9 days. There was a significant negative correlation between pain scores and sleep quality measures (sleep efficiency, immobility time, and fragmentation index).

a TKA: total knee arthroplasty.

b THA: total hip arthroplasty.

c PROM: patient-reported outcomes measure.

d HR: heart rate.

e KOOS: Knee Injury and Osteoarthritis Outcome Score.

f HOOS: Hip Disability and Osteoarthritis Outcome Score.

g pre-op: preoperative.

h N/A: not available.

i post-op: postoperative.

j TJA: total joint arthroplasty.

k VR-12: Veterans Rand 12-Item Health Survey.

l PCS: Physical Composite Score.

m SAM: step activity monitor.

n KSS: Knee Society Score.

o SF-36: 36-Item Short Form Health Survey.

p ROM: range of motion.

q OKS: Oxford Knee Score.

r JR: joint replacement.

s QoL: quality of life.

t LEFS: Lower Extremity Functional Scale.

u NRS: Numeric Pain Rating Scale.

v PDI: Pain Disability Index.

w AAS: Active Australia Survey.

x WOMAC: Western Ontario and McMaster Universities Arthritis Index.

y SRS: Surgical Recovery Scale.

z PSQI: Pittsburgh Sleep Quality Index.

aa VAS: Virtual Analog Scale.

ab OHS: Oxford Hip Score.

ac UCLA: University of California, Los Angeles Activity Scale.

ad FJS-12: Forgotten Joint Score 12.

ae NPRS: Numeric Pain Rating Scale.

af SF-12: 12-Item Short Form Health Survey.

ag HHS: Harris Hip Score.

ah BPI: Brief Pain Inventory.

ai IPO: International Pain Outcomes Questionnaire.

aj PA: physical activity.

ak ND: not defined.

al RAR: rest activity daily rhythm.

am BI: Barthel Index.

an FIM: Functional Independence Measure.

ao TUG: timed-up-and-go.

ap MWT: minute walking test.

aq TKR: total knee replacement.

ar FJS: Forgotten Joint Socre.

as P6MC: peak 6-minute consecutive cadence

at P1M: peak 1-minute cadence.

au MCID: minimal clinically important difference.

av ED: emrgency department.

aw KOOS-ADL: Knee Injury and Osteoarthritis Outcome Score-Activities if Daily Living.

## Results

### Overview

**Figure 1.**
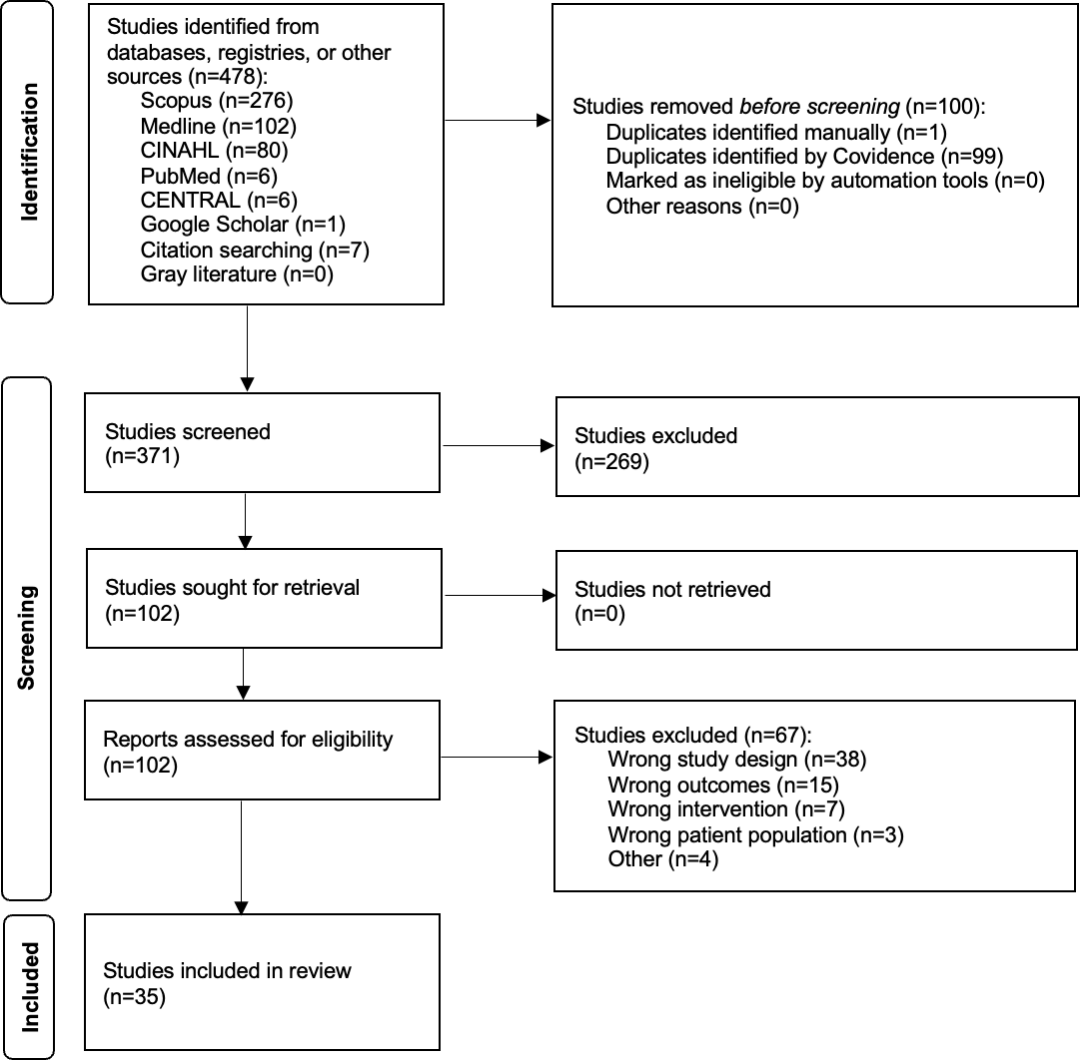
PRISMA flow diagram*.* PRISMA: Preferred Reporting Items for Systematic Reviews and Meta-Analyses.

This systematic review analyzed 35 studies that used wearable devices to assist with patient monitoring in the TJA perioperative period. These papers detail how monitoring with wearables is used in conjunction with PROMs and functional metrics in the recovery of patients undergoing TJA. The final analysis included 35 papers, including 5 RCTs and 30 nonrandomized papers (Figure 1).

### Physical Activity and Performance-Based Functional Outcomes

Of the 35 papers included in this study, 32 collected step count, making it the most common data point reported (Table 3). Another component of assessing functional outcomes in TJA is performance-based tests, such as the 6-minute walking test (6MWT), range-of-motion, or timed-up-and-go (TUG; Table 3). A total of 9 studies used both wearable device data and standardized performance-based functional outcomes in their assessments (Table 3).

### Physical Activity and PROMs

Physical activity and PROMs used were not consistent among studies (Table 3). Joint-specific PROMs, including Knee Society Score, Knee Injury and Osteoarthritis Outcome Score (KOOS), Oxford Knee Score, Hip Disability and Osteoarthritis Outcome Score (HOOS), and Harris Hip Score, were used in 21 studies. Virtual Analog Scale (VAS) was used in 4 studies [15,19,34,41] and Numerical Rating Scale for pain was used in 4 studies [15,18,24,47]. EQ-5D scores were examined in 4 studies [15,16,21,23], and 3 used Western Ontario and McMaster Universities Arthritis Index (WOMAC) [32,39,43]. The Lower Extremity Functional Scale (LEFS) was used in 2 studies [48,51]. The Veterans Rand 12-Item Health Survey was used in 2 studies [37,38], and 1 used SF-36 (36-Item Short Form Health Survey) [30].

### Sleep

Sleep as recorded by wearable devices was reported in 7 of the included studies (Table 3). Gibian et al [19] recorded sleep duration and reported a negative correlation with VAS pain scores [35]. A total of 2 additional studies [17,20,24] also report sleep duration as recorded by wearable devices. Frimpong et al [31] use a validated algorithm to report sleep data as recorded by wearables. Karas et al [44] report “sleep efficacy,” which is the proportion of minutes asleep to the total time in bed. Finally, Fallahzadeh et al [43] and Vitale et al [41] focused on multiple components of sleep, with the former including overall sleep and sleep quality and the latter recording 9 components of sleep.

### Rehabilitation Workflow

Interestingly, only 1 study used a novel application to assess patients [28]. This study demonstrated that step counts as recorded in the MoveUp application correlated with LEFS following TJA. However, the authors did not mention how this application can be used to create tailored rehabilitation protocols.

## Discussion

### Principal Findings

This systematic review found that most of the literature regarding the utility of wearable devices to monitor TJA outcomes includes step count. In addition to recording step count, these devices can record data regarding gait speed and asymmetry as well. Assessment of such metrics allows providers to evaluate gait comprehensively. Nevertheless, few studies used this capability of wearables [16,17,22,46]. Further, there is a large variability in how wearable device data is used in conjunction with other outcome measures in the current literature. Further, while ample studies include PROMs in addition to wearable data, many different PROMs are used. As demonstrated in Table 3, wearable devices have the capability of measuring multiple objective metrics. While this capability has the potential to allow holistic assessment of patient recovery, there is currently no consensus on which metrics are most valuable to record and assess in the perioperative setting. Further, the studies reviewed are not uniform in when patients are assessed, with certain groups analyzing patients days to weeks after surgery, while others continue for 6 to 12 months. The heterogeneity among these studies demonstrates that the ideal manner in which surgeons can use wearable devices for remote monitoring requires further research. We suggest that stratification regarding the reporting of perioperative data obtained by wearables, and that coupling this data with a unified PROM, will maximize the utility of wearable devices as a tool for assessing patient recovery.

The relationship between wearable-derived physical activity and PROMs, such as pain, quality of life, and self-reported function scores, was explored by 21 studies. The most common PROMs reported were joint-specific. Multiple studies found a correlation between change in step count and KOOS, KOOS JR (KOOS-joint replacement), HOOS, or HOOS JR [15,18,20,36,38,42,47,48]. Both the KOOS and HOOS were developed particularly for patients with osteoarthritis and have long been used to track progress following TJA. Such studies support the use of wearable devices as another component of comprehensive monitoring of TJA recovery, particularly since such devices allow continuous monitoring. However, the current literature does not yet suggest a data point obtained via remote wearables that necessitates intervention or that intervention improves KOOS or HOOS.

Contrastingly, some groups found that steps did not correlate with other joint-specific PROMs. Frimpong et al 2020 [31] report that there is no correlation between KOOS and an increase in physical activity. Additionally, Tang et al [27] reported that there is no correlation between accelerometry-measured activity level and HOOS JR within 3 months of TJA. Multiple studies found no correlation between step count and WOMAC [31,43] or UCLA (University of California, Los Angeles Activity Scale) activity index [35,45]. It is unclear why many studies found that step count correlated with KOOS and HOOS, but not WOMAC or UCLA activity index. One suggestion is that HOOS and KOOS have been shown to better detect changes over time, while WOMAC and UCLA are more adapted for more active populations [52,53]. Regarding the UCLA activity index, it is possible to have large increases in step number but not have an increase in activity level on this scale.

In addition to joint-specific PROMs, other measures were used. Notably, 3 studies [28,43,48] found relationships between perioperative physical activity and patients’ ability to perform everyday tasks as measured by the LEFS and Surgical Recovery Scale. Others [20,36-38] found a positive correlation between wearable device reported step count and general health and physical functioning, as measured by the Veterans Rand 12-Item Health Survey PCS (Physical Composite Score) or SF-12 (12-Item Short Form Health Survey) PCS scores.

Further, some of the reviewed studies include functional outcome measures. Performance-based tests, such as the 6MWT and TUG, allow standardized assessment of patients. These tests are normally used immediately following surgery, before patient discharge, and then repeated at certain intervals in the clinic. As such, these tests allow serial, objective evaluation of patient recovery. Paxton found that both 6MWT and step count increased 20% from baseline at 12 weeks post-TKA, while TUG improvements are present but less robust [22]. Similarly, Pechstein et al [26] found a dose-response relationship between 6MWT and device-measured physical activity. Improvements in physical activity and TUG can be seen as early as 10 days postoperatively [41], and van Diemen et al [42] found a significant correlation between activity and TUG. Step count and 6MWT both measure endurance in walking, while TUG focuses on mobility. This correlation suggests that data collected by wearable devices might serve as a surrogate for these objective assessments. In contrast to TUG and 6MWT, wearable devices allow assessments to be performed continuously and remotely. As such, patients can be assessed in between clinic visits. When patients deviate from expected progress or demonstrate setbacks, tailored protocols can be developed.

While this review found that most wearable device literature includes step count, many wearable devices have the capacity to monitor additional variables, such as balance, as well. Further investigation could examine how such parameters correlate with TUG and other performance measures.

Further, because wearable devices are used during the preoperative period, they allow providers to obtain a patient baseline before TJA. The collection of this data allows recovery trajectories to be crafted. Patients demonstrate decreases in activity, balance, and gait speed acutely postoperatively, but then return to or surpass their baseline postoperatively. In the acute postoperative setting, knowledge of patient deviation from baseline can inform discharge protocols to allow patients to return to benchmarks. As patients progress in their recovery, comparison of metrics to preoperative and immediate postoperative data informs the multidisciplinary team about patient recovery. If a patient is not meeting a step count goal, therapy can be tailored toward endurance. Contrastingly, if a patient is struggling with balance, therapy can be adjusted to focus on improving this deficit. As the number of patients who have data collected increases, there is also potential to create expected recovery trajectories for patients based on baseline preoperative data, allowing providers to provide realistic and individualized recovery expectations.

In addition to papers that focus on activity, 7 of the included studies use wearable devices to monitor sleep in the TJA perioperative period. It has been well established that sleep and pain are closely associated, and it has been further shown that higher sleep quality is associated with better acute pain relief following TKA [28,29]. Wearable devices were shown to both increase physical activity on the Active Australia Survey and reduce time in bed and sleep duration [24]. This study did not report on sleep quality as reported by the wearable device. Another study found that both daily steps and minutes slept returned to preoperative levels 3 months post THA. Again, sleep quality as measured by wearable was not reported. Karas et al [44] report a “sleep efficacy score,” which is the proportion of minutes asleep to the total time in bed. They find that reductions in sleep efficiency from baseline up to 24 weeks postoperatively, but that daily steps return to baseline 12 weeks after surgery.

Gibian et al [19] found that patient-reported sleep quality, particularly poor-quality sleep as defined by the Pittsburgh Sleep Quality Index, correlated well with VAS pain score at 30 days postoperatively. However, they found no relationship between device-recorded sleep duration and VAS. Further, they found no correlation between overall Pittsburgh Sleep Quality Index scores and device-recorded hours of sleep at 30, 60, or 90 days postoperatively. Finally, 1 study used wearable devices to monitor multiple components of sleep, including overall sleep, sleep onset, and sleep disruption. Sleep disruption increased after surgery, but overall sleep did not. Sleep duration as an isolated metric may lack sensitivity in predicting changes in a patient’s recovery timeline following TJA, but fortunately, smartwatches and activity trackers can collect more advanced measurements of sleep. Taken together, the current literature that uses wearable devices to monitor sleep in TJA is heterogeneous. Different groups report an array of variables. This underscores the immense capacity of wearable devices to track multiple domains and also highlights the fact that more uniform outcomes reporting would allow studies to be more easily compared. Further, despite sleep data being collected and reported, current literature does not report on intervening on patients with altered sleep components as reported by wearables.

Notably, 5 of the 35 reviewed studies were sponsored by Zimmer Biomet [15-19], 1 study used a mobile application the lead author created [21], and 1 study used a wearable supplied by 360 Knee Systems [20]. Redfern et al [15] report that patients in the lowest preoperative step count group have lower KOOS JR scores and demonstrate greater postoperative improvement in KOOS JR. Similarly, Pasqualini et al [18] demonstrate that step counts correlate with HOOS JR both preoperatively and at 1- and 3-month follow-up. Further, 2 industry-funded studies [16,17] suggest that PROM surpasses minimal clinically important difference earlier in recovery than activity as measured by wearables. Twiggs et al [20] found a positive correlation between postoperative step count and KOOS activities of daily living subscore. Lebleu et al [21] perform an analysis using the MoveUp application the lead author created. They find that peak cadence improved beyond minimal clinically important difference more quickly than step count in both TJA and TKA recovery. Taken together, the industry-funded studies suggest that PROMs may improve faster than wearable device measurements and that some PROMs correlate with step counts or other measures of physical activity. Further, this research demonstrates the heterogeneity in the types of wearable data reported and the PROMs used among different studies.

The major strength of this study is the comprehensive examination of existing literature. Multiple databases were searched to ensure a comprehensive review of the literature. Further, 2 authors independently extracted data and then collaborated for consensus regarding study eligibility. There was also an agreement between reviewers regarding bias assessment according to the RoB-2 or ROBINS-I tools. This review reports on the vast capabilities of wearable devices, ranging from tracking step counts and sleep to more sophisticated measures. Moreover, we report how wearable device data correlates with validated PROMs and outcome measures, emphasizing their potential to revolutionize postoperative outcome monitoring. Wearable devices also encourage patient participation in rehabilitation protocols and offer patients objective data by which to monitor their postoperative progress. The studies included for review demonstrate high patient participation and compliance.

There are several limitations to this review. As with all reviews, the quality of the reviewed studies is a limitation. Our review included only 5 RCTs, with 30 studies representing nonrandomized trials, and these have lower levels of evidence. As discussed, the heterogeneity among study methods makes direct comparisons difficult. Different wearables have different capabilities. Further, different groups collected data at different time points or had patients wear devices in different locations. While the included studies provided patients with the wearable devices, requiring patients to use a wearable is a source of bias. Patients who are familiar with these devices may have higher technological literacy, socioeconomic status, or ability to care for themselves. As such, certain populations may be excluded from these studies. Additionally, the use of a wearable device on the wrist immediately postoperatively can result in falsely low recordings if patients are using an assistive device such as a walker or cane.

Finally, we propose that remote monitoring via wearable devices provides a practical method for tracking recovery, supporting patient engagement, and detecting deviations from expected progress. Current literature does not agree on the optimal data that wearables provide or when data collection should be performed. Despite this, the studies included in this review demonstrate that variables collected via wearable devices correlate with certain PROMs, pain measures, and objective postoperative tests. As such, monitoring patients with wearables may allow a more holistic assessment of patient recovery. Further, wearable devices have the potential to allow analysis informed by baseline preoperative activity, which can enhance preoperative counseling and personalize recovery expectations. Lastly, continuous monitoring may also allow early identification of delayed recovery, enabling timely intervention and tailored rehabilitation. Further studies can explore the ideal time in recovery, variables to assess, and the combination of data collected with additional outcome measures that allow wearable devices to most effectively assess outcomes following TJA.

### Conclusions

The increasing prevalence of wearable devices presents significant opportunities for advancing postoperative care through continuous health monitoring and data collection. Remote monitoring using these devices allows surgeons to gather extensive and continuous data, which can be crucial for assessing outcomes following TJA. When integrated with a collection of PROMs and objective assessments, data from wearable devices facilitates more comprehensive patient surveillance and enables tailored interventions during the perioperative period. Early monitoring of patients in the postoperative phase enhances the timely identification of adverse events and complications, thereby allowing prompt intervention. Additionally, remote monitoring of patients has been demonstrated to improve compliance with postoperative protocols and instructions. Through rigorous evaluation and standardization of data collected, remote monitoring has the potential to elevate the quality of care and enhance the recovery experience for TJA patients, ultimately leading to improved patient outcomes and satisfaction. However, further research is needed to delineate which specific remote data points are most effective in facilitating the early identification of adverse events and in optimizing patient outcomes and satisfaction after TJA. Future studies can focus on which data provides the most valuable and complete assessment of patient recovery. As our knowledge of how wearables can be best used to assess outcomes increases, there is potential to use data provided to tailor continuous patient assessment and individualized rehabilitation protocols, customized postoperative evaluation, and early detection of deviation from norms in recovery.

## Supplementary material

10.2196/84671 Multimedia Appendix 1 Cochrane methodology for systematic reviews was used in the literature search.

10.2196/84671 Multimedia Appendix 2 Terms used in the literature search.

10.2196/84671 Multimedia Appendix 3 Risk of bias visualization tool traffic plots for included randomized control studies, nonrandomized studies, and interventions.

10.2196/84671 Checklist 1 PRISMA checklist.

## Abbreviations

HOOS: Hip Disability and Osteoarthritis Outcome Score
JR: joint replacement
KOOS: Knee Injury and Osteoarthritis Outcome Score
LEFS: Lower Extremity Functional Scale
MWT: minute walking test
PCS: Physical Composite Score
PROM: patient-reported outcome measure
RCT: randomized controlled trial
ROB-2: Cochrane’s Risk of Bias tool for randomized trials
ROBINS-I: Risk of Bias in Nonrandomized Studies of Interventions
SF-12: 12-Item Short Form Health Survey
SF-36: 36-Item Short Form Health Survey
THA: total hip arthroplasty
TJA: total joint arthroplasty
TKA: total knee arthroplasty
TUG: timed-up-and-go
UCLA: University of California, Los Angeles Activity Scale
VAS: Virtual Analog Scale
WOMAC: Western Ontario and McMaster Universities Arthritis Index
