# Supplementary figures and images for: Use of Wearable Devices to Augment Traditional Measurements of Postoperative Outcomes Following Total Joint Arthroplasty: Systematic Review
Source: JMIR Rehabil Assist Technol. 2026 Apr 17;13:e84671. doi: 10.2196/84671 (PMC13089798; doi:10.2196/84671)

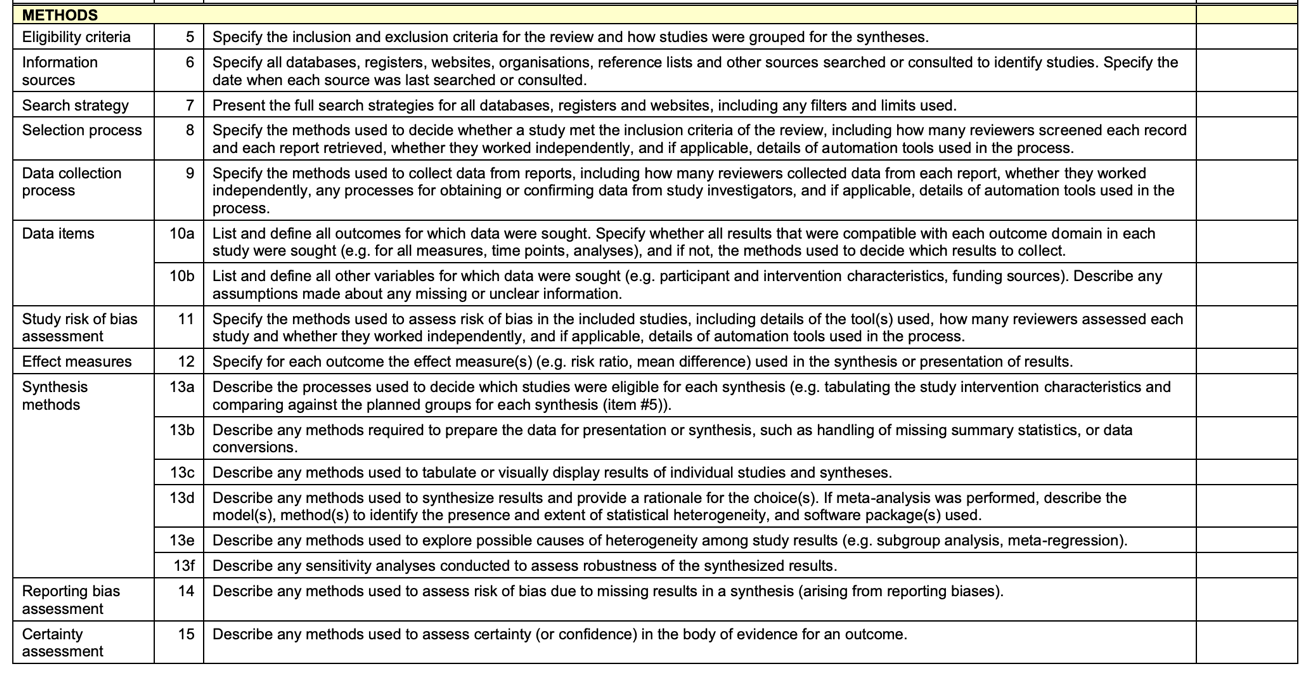

Supplement: Multimedia Appendix 1 — Cochrane methodology for systematic reviews was used in the literature search. [file rehab-v13-e84671-s001.docx]

**
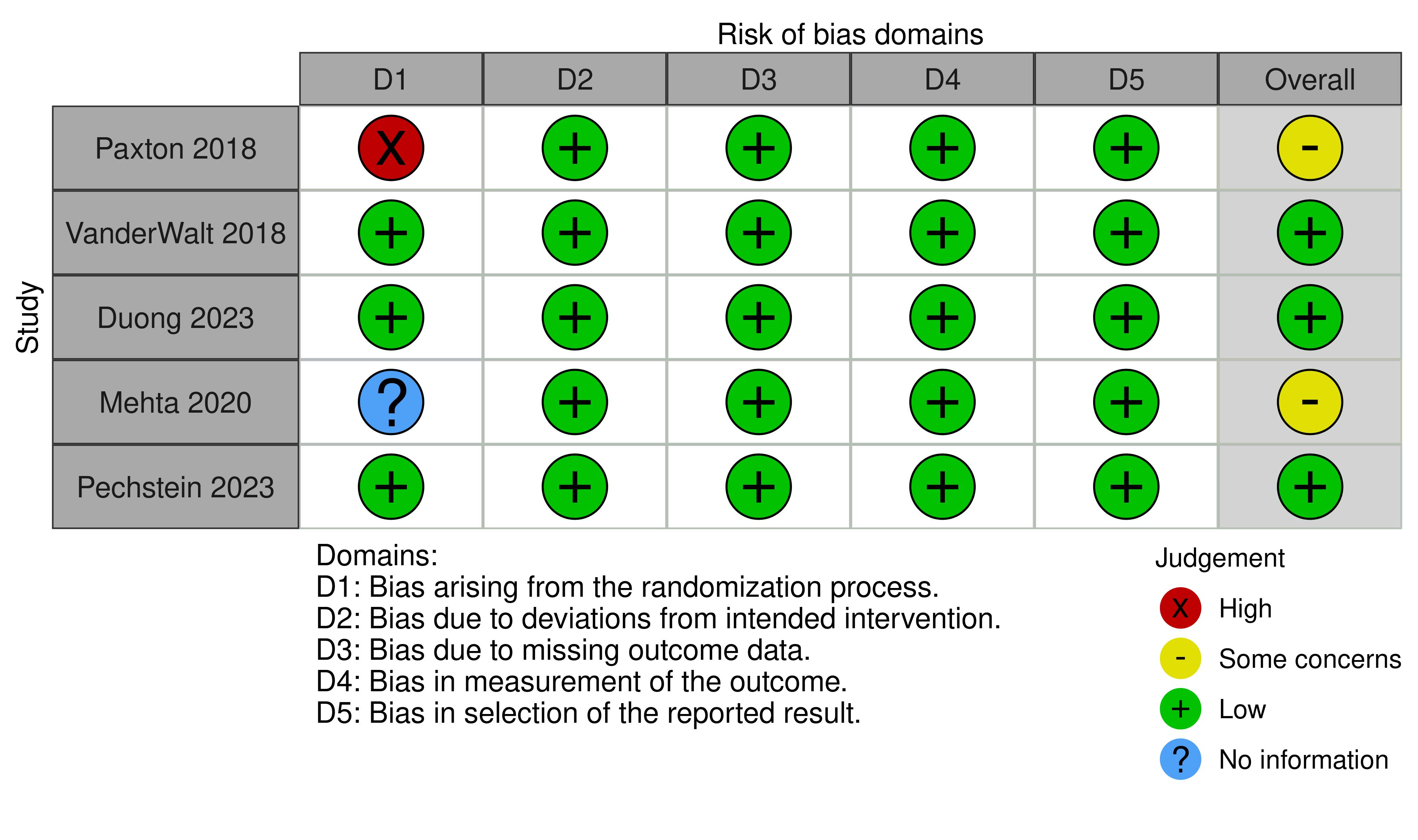

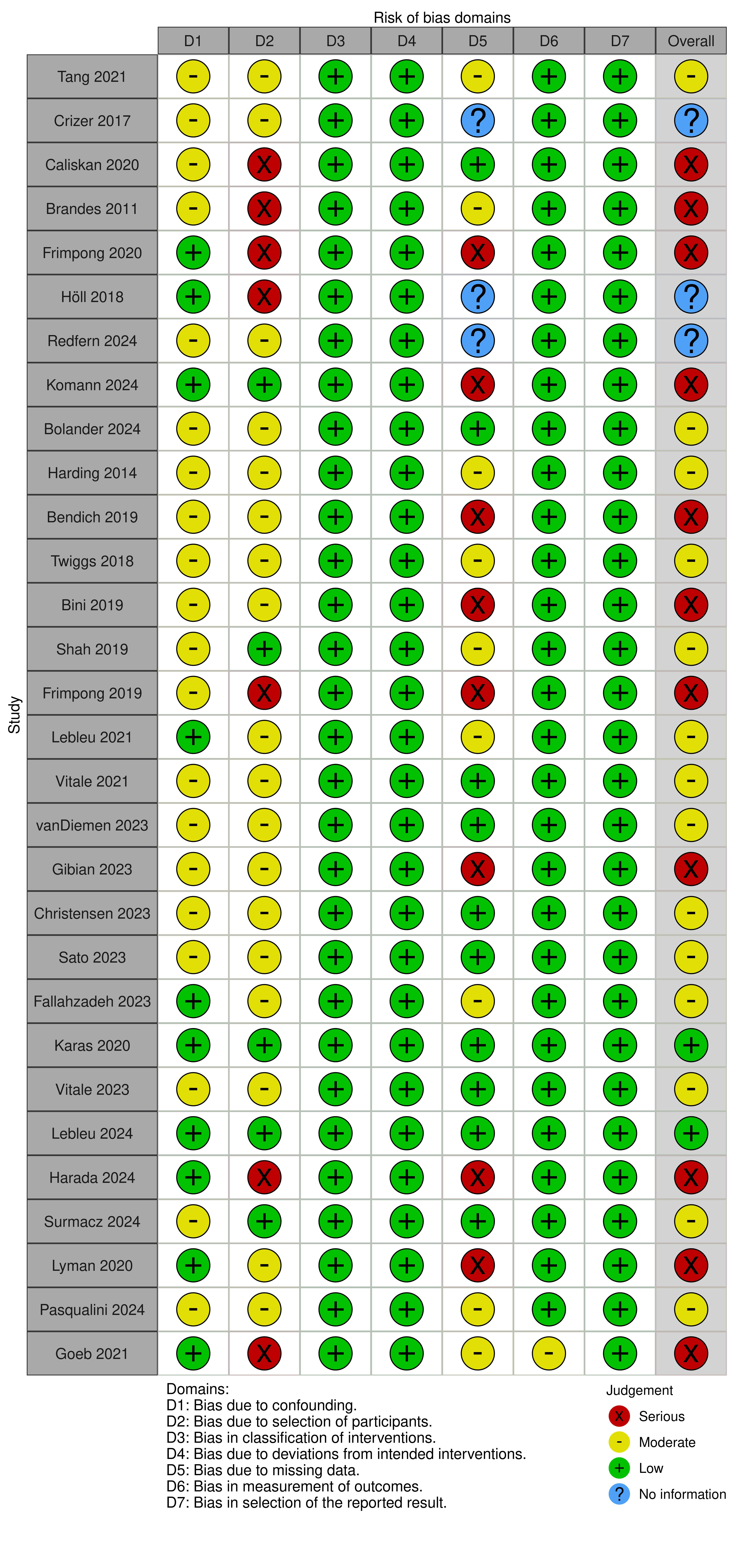
**

Supplement: Multimedia Appendix 3 — Risk of bias visualization tool traffic plots for included randomized control studies, nonrandomized studies, and interventions. [file rehab-v13-e84671-s003.docx]
