# Supplementary material for: Use of Wearable Devices to Augment Traditional Measurements of Postoperative Outcomes Following Total Joint Arthroplasty: Systematic Review
Source: JMIR Rehabil Assist Technol. 2026 Apr 17;13:e84671. doi: 10.2196/84671 (PMC13089798; doi:10.2196/84671)
Supplement: Multimedia Appendix 2 — Terms used in the literature search. [file rehab-v13-e84671-s002.docx]

Search Terms:

Wearable Electronic Devices/ OR Digital Health/ OR Monitoring, OR Mobile health (mHealth) Physiologic/ OR ((wearable ADJ2 (device* OR technolog* OR system*)) OR digital health OR physiologic* monitoring OR mobile application* OR Fitbit OR Garmin OR WHOOP or ActiGraph OR Activity trackers OR Biometric sensors OR ((Google OR Apple OR Samsung) ADJ2 watch)).ti,ab,kf.

AND

Postoperative Period/ OR exp rehabilitation/ OR exp Patient Outcome Assessment/ OR (postoperative period OR rehabilitat* OR (patient ADJ2 outcome)) OR Functional outcomes.ti,ab,kf.

AND

exp Arthroplasty, Replacement/ or (replacement arthroplasty OR joint adj2 (replacement or arthroplasty)).ti,ab,kf.
